# BACE1, the Alzheimer’s beta-secretase enzyme, in health and disease

**DOI:** 10.1186/1750-1326-8-S1-O7

**Published:** 2013-09-13

**Authors:** Robert Vassar

**Affiliations:** 1 Feinberg School of Medicine, Northwestern University, Chicago, USA

## 

The beta-amyloid (Abeta) peptide is the major constituent of amyloid plaques in Alzheimer’s disease (AD) brain and is likely to play a central role in the pathogenesis of this devastating neurodegenerative disorder. The beta-secretase, beta-site amyloid precursor protein cleaving enzyme 1 (BACE1; also called Asp2, memapsin 2), is the enzyme responsible for initiating the generation of Abeta. Thus, BACE1 is a prime drug target for the therapeutic inhibition of Abeta production for the treatment or prevention of AD. Since its discovery over 10 years ago, much has been learned about BACE1. This seminar will describe BACE1 properties, physiological functions, and dysregulation in AD. The therapeutic potential of BACE1 inhibitors for AD will also be considered. Particular focus will be placed upon our novel results demonstrating a role of BACE1 in the axon guidance of olfactory sensory neuron axons to specific odorant receptor glomeruli in the olfactory bulb and the therapeutic implications of these findings.

